# Crisis of Antimicrobial Resistance in China: Now and the Future

**DOI:** 10.3389/fmicb.2019.02240

**Published:** 2019-09-27

**Authors:** Junyan Qu, Yimei Huang, Xiaoju Lv

**Affiliations:** ^1^Center of Infectious Disease, West China Hospital, Sichuan University, Chengdu, China; ^2^College of Pharmacy, University of Florida, Gainesville, FL, United States

**Keywords:** crisis, antimicrobial resistance, China, resistance pattern, combating drug resistance

## Abstract

The crisis of antimicrobial resistance is worsening and has become a major public safety problem in China, seriously endangering human and animal health and ecological environment. Gram-negative bacterial resistance in China is severe: the related pathogens mainly include carbapenem-resistant *Acinetobacter, Pseudomonas aeruginosa* and *Klebsiella pneumoniae.* Surging antimicrobial consumption and irrational use of antimicrobials are the main causes of resistance. In China, a variety of strategies are implemented to control the antimicrobial resistance in hospitals, agriculture and environment. However, there is still a long way to go to strengthen the drug resistance surveillance, to reduce the emergence of drug-resistant bacteria, and to find new antimicrobials and therapies for drug-resistant bacteria. Controlling the antimicrobial resistance crisis takes great efforts from the whole society.

Antimicrobials have saved tens of millions of lives since penicillin was used clinically in 1940s. They have made an outstanding contribution to prolong the average life span. However, many bacteria have developed severe resistance to antimicrobials with the increased antimicrobial consumption worldwide. The development rate of bacterial resistance is much faster than that of new antimicrobials. If uncontrolled, humans will enter the “post-antibiotic era.” China is one of the top consumers of antimicrobials in the world with 1.3 billion population. Therefore, it is more challenging for China to face the antimicrobial resistance.

## The Resistance Pattern of Antimicrobials in China

Infections caused by multidrug-resistant organisms (MDROs), especially carbapenem-resistant Gram-negative bacteria often cause high mortality due to limited treatment options. Bacterial resistance data from multiple hospitals in China have been collected by China Antimicrobial Resistance Surveillance System (CARSS) (National Health and Family Planning Commission of the People’s Republic of China, 2017). In 2016, a total of 2727605 strains from 1273 hospitals were collected. Imipenem-resistant *Acinetobacter baumannii* increased from 45.8% in 2012 to 59.2% in 2016. The resistance rate of *Escherichia coli* to imipenem and third-generation cephalosporins (3GC) decreased slightly from 2.2 and 69.7% in 2012 to 1.2 and 56.3% in 2016, respectively. From 2012 to 2016, the resistance rate of *Klebsiella pneumoniae* fluctuated with the rising trend, reaching 34.5 and 8.7% in 2016 to 3GC and carbapenems, respectively. In 2016, the prevalence of carbapenem-resistant *Pseudomonas aeruginosa* (CRPA), methicillin-resistant *Staphylococcus aureus* (MRSA) and vancomycin-resistant *Enterococcus faecium* was 22.3, 34.4, and 2.0% respectively. There was no *S. aureus* with vancomycin resistance. The China Antimicrobial Surveillance Network (CHINET, 2018) began monitoring bacterial resistance nationwide since 2005. Their data helped us to understand the status and changes of bacterial resistance in China. Between 2005 and 2017, the number of bacterial strains isolated annually ranged between 22774 and 190610. Carbapenem-resistant *Acinetobacter*, of which over 90% were *A. baumannii* (CRAB), increased from 31 to 71.4%, with 60% being multidrug-resistant. *Enterobacteriaceae* were still highly sensitive to carbapenems, as the carbapenem resistance rate of most bacteria in this family was less than 10%. There was a slightly downward trend in the prevalence of *P. aeruginosa* and in its resistance rate to carbapenems (ranging from 20 to 30%, CRPA). Remarkably, there was a tenfold increase of the carbapenem-resistant *K. pneumoniae* (CRKP), from 2.4 to 24% in the past 13 years, most of which were isolated from sputum specimen. The geographical distribution of CRAB, CRKP and CRPA was mainly concentrated in central and eastern China and Yunnan Province (CHINET, 2018). On the contrary, MRSA isolates decreased from 69% in 2005 to 35.3% in 2017. The prevalence of vancomycin-resistant *Enterococci* (VRE) in China is still low (Hu et al., 2016; CHINET, 2018). In 2014, the first World Health Organization (WHO) global report on antimicrobial resistance surveillance showed that CRKP has appeared in almost all parts of the world (World Health Organization [WHO], 2014). A report on antimicrobial resistance surveillance in Europe showed that the resistance rate of *K. pneumoniae* against carbapenems was 6.1% in 2016, without a significant change from 2013 to 2016. The prevalence of CRKP in Greece was the highest, up to 61.9% (ECDC, 2016). In summary, just like the global trend, Gram-negative bacterial resistance in China is severe. In particular, the rapid growth of CRKP should draw widespread attention.

Drug resistance surveillance network for zoonotic bacteria in China was established in 2008 (Zhang et al., 2017). The key monitored strains are *E. coli, Salmonella, S. aureus, Enterococcus, Campylobacter, Streptococcus, Haemophilus parasuis*, and *Pasteurella*, etc. The serotype identification and drug resistance testing have been completed in more than 30, 000 strains of bacteria. In recent years, bacteria such as *E. coli* and *Salmonella* carried by animals have been found to be resistant to colistin. The situation is worse in poultry than in swine. *E. coli* and *Salmonella* are also highly resistant to tetracycline. The resistance rate of *E. coli* to florfenicol has been as high as 100%, and to enrofloxacin about 50–70% (Cai, 2017).

## What Drives the Antimicrobial Resistance in China?

**FIGURE 1:**
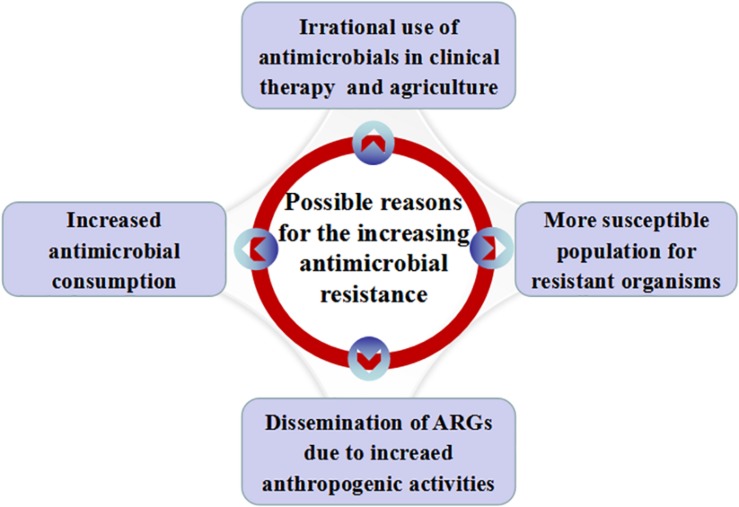
Possible reasons for the increasing antimicrobial resistance in China. Possible reasons include increased antimicrobial consumption, irrational use of antimicrobials in clinical practice and agriculture, dissemination of ARGs with the increase of anthropogenic activities and more susceptible people for resistant organisms.

Some possible reasons for the increasing antimicrobial resistance in China are illustrated in Figure 1.

First, antimicrobial consumption promotes antimicrobial resistance. A study by Klein et al. (2018) showed that antimicrobial consumption increased by 79% [2.3–4.2 billion DDDs (defined daily doses)] in China between 2000 and 2015, higher than the increase of global antimicrobial consumption, which was 65% over the 15 years. The antimicrobial consumption rate in DDDs per 1,000 inhabitants per day in China (65%) also grew faster than that of the globe (39%). In China, the higher population density, the decreased air quality due to the emission of gasoline and other fuels in the urbanization, and the high prevalence of chronic obstructive pulmonary disease (COPD) (8.6%, 99.9 million people) (Wang et al., 2018) make people become more susceptible to bacterial infections. In addition, more and more patients seek medical advice and have higher healthcare expectation with the continuous improvement of the social security system, living standards and health literacy. Therefore, the demand for antimicrobials has increased.

Secondly, irrational use of antimicrobial agents in clinical practice (especially among children) and agriculture (including livestock, aquatic products, and crops, etc.) (Zeng et al., 2017). Some general practitioners or rural doctors are unfamiliar with the principles and methods for the rational application of antimicrobials, they may wrongly prescribe antimicrobials in the ways such as incorrect dosing, topical application of systemic antimicrobials, and improper antimicrobial prophylaxis, etc. Most often, patients with viral infections (flu or common cold) may be prescribed antimicrobials. A study from Poland also showed increased antimicrobial consumption in viral infection season (Ciszewski et al., 2017). Financial incentives, such as mark-ups on drug price, is considered to be the main driver of over-prescribing in China (Qiao et al., 2018). Many people have low literacy about antimicrobials, and they pursue antimicrobials through a pharmacy without prescription (online or on site) (Wang et al., 2016). Antimicrobials are widely used in livestock as prophylactic and therapeutic agent for infections and as growth promoters. Antimicrobial use in livestock is even slightly higher than in humans (Zhang et al., 2015). Antimicrobials have contaminated the food and drinking water supply in China because a large number of antimicrobials are used improperly in livestock in rural China (Hao et al., 2015). In 2015, a survey on the antimicrobial body burden of Chinese schoolchildren found that 58.3% of 1064 urine samples were tested positive for antimicrobials, and that the contaminated environment and food may be the main sources of exposure (Wang et al., 2015). This may have induced bacterial resistance and unbalanced flora distribution, damaged the immune function and nervous system, and produced other adverse drug reactions.

Thirdly, antibiotic resistance genes (ARGs) are a natural component of all environments. However, anthropogenic activities have led to the dissemination of ARGs as an emerging environmental contaminant (Zhu et al., 2017; Qiao et al., 2018). Now, ARGs are widely distributed in China in environments including clinical areas, surface water, animal wastes, sewage treatment plant effluents and soils (Qiao et al., 2018). Antimicrobials and ARGs can spread among the environment, humans and animals, which is closely associated with the increasing prevalence of antimicrobial resistance and is threatening human health. A recent study even found that smog metagenomes in Beijing contained multiple carbapenem-resistant genes. The relative abundance was similar to that in the human gut and sewage (Pal et al., 2016).

Fourthly, bacterial defense dysfunction occurs in some populations as a result of the aging Chinese society, the long-term use of steroids and immunosuppressants, the extensive development of bone marrow and organ transplantation, the increased number of invasive treatments, and the prevalence of acquired immunodeficiency syndrome (AIDS). In these populations, their demand for antimicrobials is greater and they are more likely to become critically ill. They are more susceptible to resistant organisms because of risk factors including ICU admission, being elderly, indwelling devices (such as central venous catheters, catheters, endotracheal tubes) and invasive procedures (Kaye and Pogue, 2015). It is very difficult to treat infections caused by multi-drug resistant strains in these patients. Maybe it is also one of the reasons for the severe bacterial resistance in China.

## How to Deal With the Antimicrobial Resistance in China?

The theme for World Health Day 2011 was “combating drug resistance: no action today, no cure tomorrow,” which reinforced all countries around the world to take more active actions against bacterial resistance. The 2015 World Health Assembly adopted the global action on antimicrobial resistance. The 2016 UN High Level Meeting on antimicrobial resistance and the G20 Summit have made strong commitments to control antimicrobial resistance. What have we done? What else do we need to do?

The Chinese government attaches great importance to the issue of antimicrobial resistance and takes multiple measures to strengthen the antimicrobial stewardship. A series of documents were promulgated such as Administrative Measures for the Clinical Use of Antibacterial Drugs (2012) (Ministry of Health of the PRC, 2012), Antimicrobial Management will be Enhanced in Multiareas (2015) (National Health and Family Planning Commission of the PRC, 2015a), Five Year Action Plan for the Comprehensive Management of Veterinary Drugs in China (2015–2019) (Ministry of Agriculture and Rural Affairs of the PRC (2015–2019), 2015), National Action Plan to Contain Antimicrobial Resistance (2016-2020) (National Health and Family Planning Commission of the PRC (2016–2020), 2016), and Work Program for the Reduction of the Use of Antimicrobials in Animals (2018–2021) (Ministry of Agriculture and Rural Affairs of the People’s Republic of China (2018–2021), 2018), etc. These documents fully demonstrate that the state will strengthen supervision over the manufacture, circulation and use of antimicrobials and support the development of new antimicrobials. Therefore, regular training on rational use of antimicrobials, enhancement of antimicrobial stewardship, and strict implementation of infection control measures, especially hand hygiene have been carried out in the medical institutions at all levels to reduce the unnecessary consumption of antimicrobials and to delay the emergence and spread of resistant bacteria.

The management of clinical use of antimicrobials in China has gone through several stages: promulgation and implementation of guidelines for clinical use of antimicrobials (2004), specialized rectification (2011–2013), continuous improvement (2014–2017) with updated guidelines for clinical use of antimicrobials (2015), and refined and standardized management training since 2018 (Ministry of Health of the PRC, 2004; National Health and Family Planning Commission of the PRC, 2011, 2015b; National Health Commission of the People’s Republic of China, 2018). These initiatives have achieved remarkable results through continuous efforts in recent years. According to the data from the Center for Antibacterial Surveillance (National Health and Family Planning Commission of the People’s Republic of China, 2017), the use of antimicrobials in outpatient settings decreased from 17.2% in 2011 to 10.3% in 2016, in inpatient settings from 59.4% in 2011 to 37.5% in 2016. Antimicrobial drug use intensity decreased from 85.10 DDD in 2005 to 50.03 DDD in 2016. However, the sales of antimicrobials to children increased from ¥5 billion (US$ 781 million) in 2005 to ¥12 billion ($1.87 billion) in 2015 according to an analysis of Chinese children’s drug market in 2017. The National Health Commission issued the document on May 10, 2018 to strengthen the clinical application and management of antimicrobials for key populations such as children (National Health Commission of the People’s Republic of China, 2018).

**FIGURE 2:**
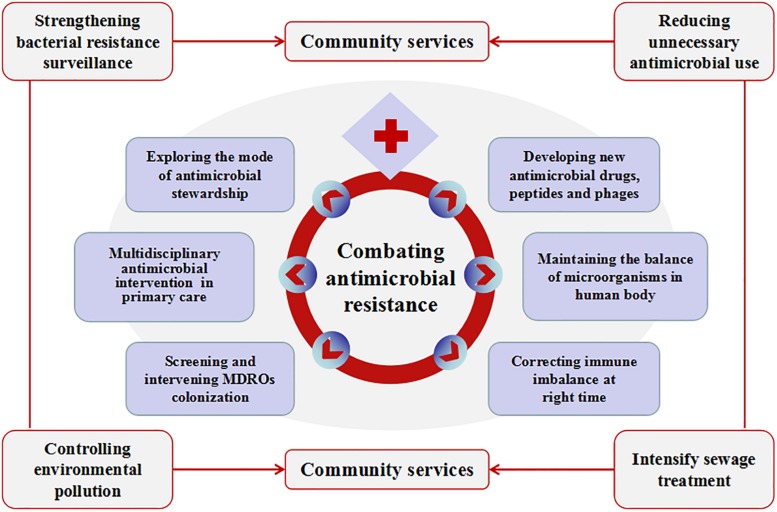
Measures to be taken to combat antimicrobial resistance in China. Combating antimicrobial resistance, we need to carry out the work from community service and medical institutions. Community service includes strengthening bacterial resistance surveillance, reducing unnecessary antimicrobial use, controlling environmental pollution and intensify sewage treatment. Other measures that can be implemented in medical institutions include exploring the mode of antimicrobial stewardship, multidisciplinary antimicrobial consults in primary health care; screening and intervening multidrug-resistant organisms’ colonization; developing new antimicrobial drugs, peptides and phages; maintaining the balance of microorganisms in the human body and correcting immune imbalance at the right time.

All the above shows that China’s firm determination to fight against bacterial resistance, but there are still many areas to be improved. Figure 2 is a schematic diagram. First, bacterial resistance surveillance in China is limited by its scope and uneven levels, especially antimicrobial resistance surveillance of animal-derived bacteria. Surveillance network of antimicrobial utilization and resistance patterns from pharmacies, clinics, hospitals, environment, agriculture and animal husbandry should be established at both local and national levels. Efforts should be made to strengthen the “big data” of bacterial resistance in all these sources, and to further address the intrinsic connections hidden behind the data. Nowadays, the development of whole-gene sequencing (WGS) technology can help researchers predict antimicrobial resistance more efficiently, thus assisting in clinical diagnosis and treatment decisions. WGS-powered online database, which developed by the Chinese scientists called BacWGSTdb, aims to pioneer the movement of WGS from proof-of-concept studies to routine use in clinical microbiology laboratory, offers a rapid and convenient platform to analyze epidemiological outbreak and the phylogeny of the bacterial genome, so as to provide information guarantee and decision-making support for prevention and control of infectious disease outbreaks and major bio-safety accidents (Ruan and Feng, 2016; Quainoo et al., 2017; Rossen et al., 2018). Second, there is still irrational use of antimicrobials in healthcare (especially among children) and agriculture (especially in animal husbandry). The standards and regulations for environmental discharge of antimicrobials still need to be improved. Chinese government has launched some measures to halt financial incentives such as the separation of prescription sales from physicians’ income and the “zero mark-up” policy on drug sale. There is a huge amount of antimicrobial consumption in animal husbandry. Animal breeders, especially farmers, lack understanding of antimicrobial resistance and its hazards. Animal-use antimicrobials should be purchased for treatment with prescriptions from a veterinarian. Reducing the use of unnecessary antimicrobials in livestock farms has not significantly harmed animal health or farmers’ incomes (Dierikx et al., 2016). Strengthen scientific breeding and management of livestock and poultry should help to reduce antimicrobial consumption in animals. Public awareness of the prevention and control of the bacterial resistance needs to be gradually raised. Third, it is necessary to continue to strictly control the sources of antimicrobial pollution from various aspects such as non-therapeutic use of antimicrobials and discharge of antimicrobial-containing sewage. The control of waste residue in antimicrobial industry should be taken into concern. Advanced oxidation processes (AOPs) might be used to improve the removal of ARGs in municipal sewage effluent (Zhang et al., 2016). ARG metagenomic data make it possible to track ARG contamination sources (Li et al., 2018), which is important for the control of ARG contamination. Fourth, we should actively explore the mode of antimicrobial stewardship suitable for the institutional development. Improve the organizational structure of antimicrobial clinical application, clarify the responsibilities and strengthen the fine management. Provide regular training and education for the rational application of antimicrobials. Establish a long-term mechanism of multidisciplinary case discussion and a multidisciplinary consulting team that consists of department of infectious diseases, clinical microbiological laboratory, department of pharmacy and department of nosocomial infection control. Under the support of information technology, strengthen the stratified management of antimicrobials, dynamically monitor the use of antimicrobial agents, evaluate the suitability and rectification of antimicrobial use. Transfer the management of antimicrobials from administrative intervention to multidisciplinary collaboration focusing on competency and patient-centered care. In addition, strengthen international cooperation is another important way to better implement antimicrobial stewardship and to stem the tide of antimicrobial resistance (Hoffman et al., 2015). Fifth, at present, many policies and training of antimicrobial agents in China are concentrated in hospitals above the level-II, but about 60% of patients in the country are treated in community hospitals/rural hospitals (level-I). To tackle this mismatch, multi-disciplinary intervention methods such as professional training in the diagnosis and treatment of infectious diseases, use of social media tool such as WeChat and on-site training platforms organized by tertiary hospitals are offered to healthcare professionals and patients in community hospitals/rural hospitals to create a model for antimicrobial intervention in primary health care institutions in China. Sixth, active screening and enhanced interventions for MDRO’s colonization in high-risk patients should be an important way to reduce drug-resistant bacterial infections. Strict isolation enhanced environmental disinfection and hand hygiene should be implemented in MDRO-colonized patients. Currently, different de-planting measures have been adopted according to different colonizing bacteria and different planting sites. Oral cleaning is performed with chlorhexidine, nasal cleaning is performed with iodide or mupirocin: feces with intestinal drug-resistant bacteria are processed separately. Minimizing hospital length of stay and reducing the conversion rate from colonization to infections may be one of the most important infection control measures. Seventh, the scientific challenges are the main problems in the development of new antimicrobial agents. Low profit for pharmaceutical companies on research and development of new drugs is another obstacle. The use of antimicrobial peptides as adjuvants to antimicrobials could probably slow down the development of drug resistance (Lázár et al., 2018). New research suggested that immunomimetic designer cells might be used to cure resistant bacterial infections in the future (Liu et al., 2018). Promoting the development and use of vaccines may reduce the demand for antimicrobials, especially in animal feed. The rapid development of DNA sequencing and artificial intelligence (AI) makes it possible to screen phages rapidly and efficiently. Phage therapy is promising to be a powerful weapon against super drug-resistant bacteria. Eighth, there are trillions of bacteria and other microorganisms in the human body. They coexist in symbiosis with human beings. Many factors, such as irrational use of antimicrobials, unhealthy diet or long-term stress and anxiety could destroy the balance of microorganisms and lead to various diseases. This is consistent with the Traditional Chinese Medicine rationale “When there is sufficient healthy qi inside, pathogenic factors have no way to invade”. If people live in harmony with bacteria, fungi or viruses, people are less likely to get sick. In recent years, fecal microbiota transplantation (FMT) and probiotics are increasingly used in the treatment of various diseases such as *Clostridium difficile* infection, inflammatory bowel disease, constipation, diabetes and obesity, etc. (Rossen et al., 2015). These methods are all based on the restoration of dysregulated intestinal flora to cure the diseases. Maybe it will provide new insights for reducing the emergence of resistant bacteria and finding new treatments in the future. Ninth, immunity dysfunction, especially immune suppression during late stage of infection plays an important role in the development and prognosis of severe infections (Delano and Ward, 2016). If the immune status of patients with severe infections can be accurately monitored and effectively intervened, the prognosis of these patients could be significantly improved, then the dilemma of “no drug available” could be changed. There will be new discoveries and breakthroughs in the study of infection and immune balance.

In short, antimicrobial resistance in China must be curbed. It calls for the power of the whole society to control the antimicrobial resistance now and in the future. Antimicrobials are precious resources for us humans, and it is everyone’s responsibility to protect them.

## Author Contributions

XL and JQ conceived and designed the study. JQ and YH wrote the manuscript. All authors reviewed and approved the final version of the manuscript.

## Conflict of Interest

The authors declare that the research was conducted in the absence of any commercial or financial relationships that could be construed as a potential conflict of interest.
